# The sense of coherence scale: psychometric properties in a representative sample of the Czech adult population

**DOI:** 10.1186/s40359-024-01805-7

**Published:** 2024-05-26

**Authors:** Martin Tušl, Ivana Šípová, Martin Máčel, Kristýna Cetkovská, Georg F. Bauer

**Affiliations:** 1 Division of Public and Organizational Health, Center of Salutogenesis, Epidemiology, Biostatistics and Prevention Institute, University of Zurich, Hirschengraben 84, Zurich, 8001 Switzerland; 2 Department of Psychology, Faculty of Arts, Charles University, Prague, Czech Republic

**Keywords:** Salutogenesis, Sense of coherence, Confirmatory factor analysis, Psychometrics, Czech adult population, Mental health

## Abstract

**Background:**

Sense of coherence (SOC) is a personal resource that reflects the extent to which one perceives the world as comprehensible, manageable, and meaningful. Decades of empirical research consistently show that SOC is an important protective resource for health and well-being. Despite the extensive use of the 13-item measure of SOC, there remains uncertainty regarding its factorial structure. Additionally, a valid and reliable Czech version of the scale is lacking. Therefore, the present study aims to examine the psychometric properties of the SOC-13 scale in a representative sample of Czech adults.

**Methods:**

An online survey was completed by 498 Czech adults (18–86 years old) between November 2021 and December 2021. We used confirmatory factor analysis to examine the factorial structure of the scale. Further, we examined the variations in SOC based on age and gender, and we tested the criterion validity of the scale using the short form of the Mental Health Continuum (MHC) scale and the Generalized Anxiety Disorder (GAD) scale as mental health outcomes.

**Results:**

SOC-13 showed an acceptable one- and three-factor fit only with specified residual covariance between items 2 and 3. We tested alternative short versions by systematically removing poorly performing items. The fit significantly improved for all shorter versions with SOC-9 having the best psychometric properties with a clear one-factorialstructure. We found that SOC increases with age and males score higher than females. SOC showed a moderately strong positive correlation with MHC, and a moderately strong negative correlation with GAD. These findings were similar for all tested versions supporting the criterion validity of the SOC scale.

**Conclusion:**

Our findings suggest that shortened versions of the SOC-13 scale have better psychometric properties than the original 13-item version in the Czech adult population. Particularly, SOC-9 emerges as a viable alternative, showing comparable reliability and validity as the 13-item version and a clear one-factorial structure in our sample.

**Supplementary Information:**

The online version contains supplementary material available at 10.1186/s40359-024-01805-7.

## Background

Sense of coherence (SOC) was introduced by the sociologist Aaron Antonovsky as the main pillar of his salutogenic theory, which explains how individuals cope with stressors and stay healthy even in case of adverse life situations [1]. SOC is a personal resource defined as a global orientation to life determining the degree to which one perceives life as comprehensible, manageable, and meaningful [2]. A strong SOC enables individuals to cope with stressors and manage tension, thus moving to the ease-end of the ease/disease continuum [2, 3]. A person’s strength of SOC can be measured with the Orientation to Life Questionnaire commonly referred to as the SOC scale [4]. The original version is composed of 29 items (SOC-29) and Antonovsky recommended 13 items for the short version of the scale (SOC-13). To date, both versions of the scale have been used across diverse populations in at least 51 languages and 51 countries [5]. Studies have consistently shown that SOC correlates strongly with different health and well-being outcomes [6, 7] and quality of life measures [8]. In the context of the recent COVID-19 pandemic, SOC has been identified as the most important protective resource in relation to mental health [9]. Regarding individual differences, SOC has been shown to strengthen over the life course [10], males usually score higher than females [11], and some studies indicate that SOC increases with the level of education [12]. However, despite the extensive evidence on the criterion validity of the scale, there is still a lack of clarity about its underlying factor structure and dimensionality.

The SOC scale was conceptualized as unidimensional suggesting that SOC in its totality, as a global orientation, influences the movement along the ease/dis-ease continuum [2]. However, the structure of the scale is rather multidimensional as each item is composed of multiple elements. Antonovsky developed the scale according to the facet theory [13, 14] which assumes that social phenomena are best understood when they are seen as multidimensional. Facet theory involves the construction of a mapping sentence which consists of the facets and the sentence linking the facets together [15]. The SOC scale is composed of five facets: (i) the response mode (comprehensibility, manageability, meaningfulness); (ii) the modality of stimulus (instrumental, cognitive, affective), (iii) its source (internal, external, both), (iv) the nature of the demand it poses (concrete, diffuse, affective), (v) and its time reference (past, present, future). For example, item 3 “Has it happened that people whom you counted on disappointed you?” is a *manageability* item that can be described with the mapping sentence as follows: "Respondent X responds to an *instrumental* stimulus (“counted on”), which originated from the *external* environment (“people”), and which poses a *diffuse* demand (“disappointed”) being in the *past* (“has it happened”)." Although each item can be categorized along the SOC component comprehensibility, manageability, or meaningfulness, the items also share elements from the other four facets with items within the same, but also within the other SOC components (see 2, Chap. 4 for details). As Antonovsky states [2, p. 87]: “The SOC facet pulls the items apart; the other facets push them together.”

Thus, the multi-facet nature of the scale can create difficulties in identifying the three theorized SOC components using statistical methods such as factor analysis. In fact, both the unidimensional and the three-dimensional SOC-13 rarely yield an acceptable fit without specifying residual covariance between single items (see 5 for an overview). This has been further exemplified in a recent study which examined the dimensionality of SOC-13 using a network perspective. The authors were unable to identify a clear structure and concluded that SOC is composed of multiple elements that are deeply linked and not necessarily distinct [16]. As a result, several researchers have suggested modified [17] or abbreviated versions of the scale, such as SOC-12 [18, 19], SOC-11 [20–22], or SOC-9 [23], which have empirically shown a better factorial structure. This prompts the general question, whether an alternative short version should be preferred over the 13-item version. In fact, looking into the original literature [2], it is not clear why Antonovsky chose specifically these 13 items from the 29-item scale. We will address this question with the Czech version of the SOC-13 scale.

### Salutogenesis in the Czech Republic

Salutogenesis and the SOC scale were introduced to the Czech audience in the early 90s by a Czech psychologist Jaro Křivohlavý. His work included the Czech translation of the SOC-29 scale [24] and the application of the concept in research on resilience [25] and behavioral medicine [26]. Unfortunately, the early Czech translation of the scale by Křivohlavý is not available electronically, nor could we locate it in library repositories. Later studies examined SOC-29 in relation to resilience [27, 28] and self-reported health [29, 30], however, it is not clear which translation of SOC-29 the authors used in the studies. A new Czech translation of the SOC-13 scale has recently been developed by the authors of this paper to examine the protective role of SOC for mental health during the COVID-19 crisis [31]. In line with earlier studies [9], SOC was identified as an important protective resource for individual mental health. This recent Czech translation of the SOC-13 scale [31] is the subject of the present study.

## Present study

Our study aims to investigate the psychometric properties of the SOC-13 scale within a representative sample of the Czech adult population. Specifically, we will examine the factorial structure of the SOC-13 scale to understand its underlying dimensions and evaluate its internal consistency to ensure its reliability as a measure of SOC. Additionally, we aim to assess criterion validity by examining the scale’s association with established measures of positive and negative mental health outcomes - the Mental Health Continuum [32] and Generalized Anxiety Disorder [33]. We anticipate a strong correlation between these measures and the SOC construct [6]. Furthermore, we will investigate demographic variations in SOC, considering factors such as age, gender, and education. Understanding these variations will provide valuable insights into the applicability of the SOC-13 scale across different population subgroups. Finally, we will explore whether alternative short versions of the SOC scale should be preferred over the 13-item version. This analysis will help determine the most efficient version of the SOC scale for future research.

## Methods

### Study design and data collection

Our study design is a cross-sectional online survey of the Czech adult population. We contracted a professional agency DataCollect (www.datacollect.cz) to collect data from a representative sample for our study. Participants were recruited using quota sampling. The inclusion criteria were: being of adult age (18+), speaking the Czech language, and having permanent residence in the Czech Republic. Exclusion criteria related to study participation were predetermined to minimize the risk of biases in the collected data. The order of items in all measures was randomized and we implemented two attention checks in the questionnaire (e.g. “Please, choose option number 2”). Participants were excluded if they did not finish the survey, completed the survey in less than five minutes, did not pass the attention checks, or gave the same answer to more than 10 consecutive items. Data collection was conducted via the online platform Survey Monkey between November 2021 and December 2021.

### Translation into the Czech language

Translation of the SOC scale was carried out by the authors of the paper with the help of a qualified translator. We followed the translation guidelines provided on the website of the Society for Research and Theory on Salutogenesis (www.stars-society.org), where the original English version of the SOC scale is available for download. Two translations were conducted independently, then compared and checked for differences. Based on this comparison, the agreed version of the scale was back translated into English by a Czech-English translator. The final version was checked for resemblance to the original version in content and in form. Although we used only the short version of the scale in our study (i.e., SOC-13), the translation included the full SOC-29 scale. The Czech translation of the full SOC scale is available as supplementary material.

### Measures

*Sense of coherence.* We used the short version of the Orientation to Life Questionnaire [3] to assess SOC. The measure consists of 13 items evaluated on a 7-point Likert-type scale with different response options. Five items measure comprehensibility (e.g., “Does it happen that you experience feelings that you would rather not have to endure?”), four items measure manageability (e.g., “Has it happened that people whom you counted on disappointed you?”), and four items measure meaningfulness (e.g., “Do you have the feeling that you really don’t care about what is going on around you?”). In our sample, Cronbach’s alpha for the full scale was α = 0.88, for comprehensibility α = 0.76, manageability α = 0.72, and meaningfulness α = 0.70.

*Mental health continuum - short form* (MHC-SF; 32). This scale consists of 14 items that capture three dimensions of well-being: (i) emotional (e.g. “During the past month, how often did you feel interested in life?”); (ii) social (e.g. “During the past month, how often did you feel that the way our society works makes sense to you?”); (iii) psychological (e.g. “During the past month, how often did you feel confident to think or express your own ideas and opinions?”). The items assess the experiences the participants had over the past two weeks, the response options ranged from 1 (never) to 6 (every day). Internal consistency of the scale was α = 0.90.

*Generalized anxiety disorder* (GAD; 33). The scale consists of seven items that measure symptoms of anxiety over the past two weeks. Sample items include, e.g. “Over the past two weeks, how often have you been bothered by the following problems?” (i) “feeling nervous, anxious, or on edge”, (ii) “worrying too much about different things”, (iii) “becoming easily annoyed or irritable”. The response options ranged from 0 (not at all) to 3 (almost every day). Internal consistency of the scale was α = 0.92.

*Sociodemographic characteristics* included age, gender, and level of education (i.e., primary/vocational, secondary, tertiary).

### Analytical procedure

Data analysis was conducted in R [34]. For confirmatory factor analysis, we used the *cfa* function of the *lavaan* package 0.6–16 [35]. We compared a one-factor model of SOC-13 to a correlated three-factor model (correlated latent factors comprehensibility, manageability, and meaningfulness) and a bi-factor model (general SOC dimension and specific dimensions comprehensibility, manageability, meaningfulness). Based on the empirical findings we further assessed the fit of alternative shorter versions of the SOC scale. We assessed the model fit using the comparative-fit index (CFI), Tucker-Lewis index (TLI), root mean square error of approximation (RMSEA), and standardized root mean square residual (SRMR) with the conventional cut-off values. The goodness-of-fit values for CFI and TLI surpassing 0.90 indicate an acceptable fit and exceeding 0.95 a good fit [36]. A value under 0.08 for RMSEA and SRMR indicates a good fit [37]. Nested models were compared using chi-square difference tests and the Bayesian Information Criterion (BIC). Models with lower BIC values should be preferred over models with higher BIC values [38]. All models were fitted using maximum likelihood estimation.

Further, we used the *cor* function of the *stats* package 4.3.2 [34] for Pearson correlation analysis to explore the association between SOC-13 and age, the *t.test* function of the same package for between groups t-test for differences based on gender, and the *aov* function with posthoc tests of the same package for one-way between-subjects ANOVA to test for differences based on level of education. To examine the criterion validity of the scale, we used the *cor* function for Pearson correlation analysis to examine the associations between SOC-13, MHC-SF, and GAD. We conducted the same analyses for the alternative short versions of the scale.

## Results

### Participants

The median survey completion time was 11 min. In total, 676 participants started the survey and 557 completed it. Of those, 56 were excluded due to exclusion criteria. One additional respondent was excluded because of dubious responses on demographic items (e.g., 100 years old and a student), and two respondents were excluded for not meeting the inclusion criteria (under 18 years old). The final sample included *N* = 498 participants. Of those, 53.4% were female, the average age was 49 years (*SD* = 16.6; range = 18–86), 43% had completed primary, 35% secondary, and 22% tertiary education. The sample is a good representation of the Czech adult population^1^ with regard to gender (51% females), age (*M* = 50 years), and education level (44% primary, 33% secondary, 18% tertiary). Representativeness was tested using chi-squared test which yielded non-significant results for all domains.

### Descriptive statistics

In Table 1, we present an inter-item correlation matrix along with skewness, kurtosis, means and standard deviations of single items for SOC-13. Item correlations ranged from *r* = 0.07 (items 2 and 4) to *r* = 0.67 (items 8 and 9). Strong and moderately strong correlations were found also across the three SOC dimensions (e.g., *r* = 0.77 comprehensibility and manageability).

**Table 1:** Means, standard deviations, skewness, kurtosis, and zero-order correlations of the SOC-13 items

Item (dimension)	M	SD	Skew.	Kurt.	1	2	3	4	5	6	7	8	9	10	11	12	13	14	15	16	17
1. Item 1 (Me)	5.02	1.78	-0.74	2.61	1																
2. Item 2 (Co)	3.73	1.68	0.18	2.17	0.10*	1															
3. Item 3 (Ma)	3.96	1.52	0.08	2.05	0.25	0.51	1														
4. Item 4 (Me)	5.26	1.56	-0.91	3.45	0.26	0.07^ns^	0.20	1													
5. Item 5 (Ma)	4.76	1.82	-0.46	2.15	0.19	0.32	0.45	0.34	1												
6. Item 6 (Co)	4.97	1.71	-0.62	2.51	0.22	0.24	0.27	0.44	0.43	1											
7. Item 7 (Me)	4.89	1.36	-0.45	2.84	0.23	0.15	0.24	0.49	0.33	0.35	1										
8. Item 8 (Co)	4.67	1.81	-0.39	2.07	0.27	0.25	0.35	0.46	0.46	0.63	0.44	1									
9. Item 9 (Co)	4.27	1.97	-0.19	1.82	0.18	0.29	0.38	0.37	0.48	0.52	0.39	0.67	1								
10. Item 10 (Ma)	4.21	1.58	-0.28	2.27	0.21	0.30	0.38	0.34	0.33	0.44	0.39	0.50	0.49	1							
11. Item 11 (Co)	4.50	1.31	-0.59	2.79	0.13**	0.11**	0.08^ns^	0.29	0.22	0.41	0.19	0.36	0.38	0.28	1						
12. Item 12 (Me)	4.99	1.83	-0.70	2.50	0.21	0.23	0.35	0.55	0.44	0.57	0.47	0.60	0.53	0.47	0.39	1					
13. Item 13 (Ma)	5.00	1.73	-0.56	2.28	0.22	0.28	0.34	0.44	0.39	0.54	0.35	0.61	0.59	0.46	0.39	0.53	1				
14. Comprehensibility	4.43	1.22	-0.20	2.50	0.26	0.53	0.46	0.46	0.55	0.79	0.44	0.83	0.82	0.57	0.58	0.65	0.68	1			
15. Manageability	4.48	1.23	-0.27	2.55	0.29	0.47	0.72	0.45	0.75	0.58	0.45	0.65	0.66	0.73	0.33	0.61	0.75	0.77	1		
16. Meaningfulness	5.04	1.18	-0.53	2.97	0.61	0.19	0.36	0.78	0.45	0.55	0.72	0.62	0.51	0.49	0.35	0.79	0.53	0.63	0.62	1	
17. SOC	4.63	1.08	-0.29	2.60	0.42	0.47	0.58	0.63	0.66	0.73	0.59	0.80	0.77	0.67	0.49	0.77	0.74	0.92	0.90	0.83	1

Note. *N* = 498; all correlations are significant at *p* < 0.001, if not indicated otherwise, *p < 0.05, **p < 0.01, ns = not significant.

### Confirmatory factor analysis

#### SOC-13

A one-factor model showed inadequate fit to the data [χ2(65) = 338.2, CFI = 0.889, TLI = 0.867, RMSEA = 0.092, SRMR = 0.062]. Based on existing evidence [6], we specified residual covariance between items 2 and 3 and tested a modified one-factor model. The model showed an acceptable fit to the data [χ2(64) = 242.6, CFI = 0.927, TLI = 0.911, RMSEA = 0.075, SRMR = 0.050], and it was superior to the one-factor model (Δχ2 = 95.5, Δ*df* = 1, *p* < 0.001).

**Fig. 1:**
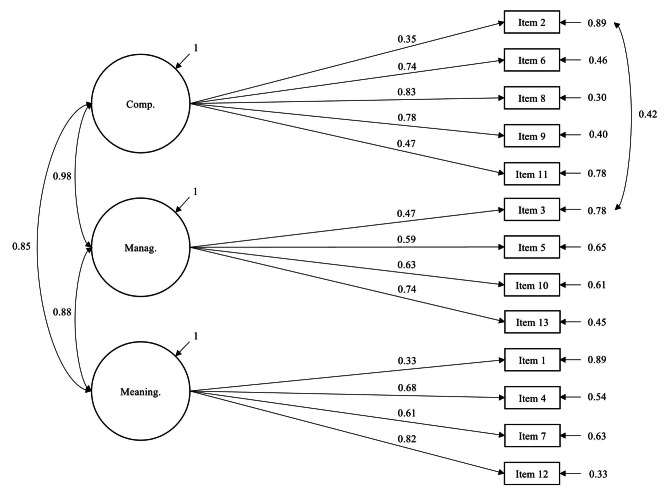
Correlated three-factor model of SOC-13 with residual covariance between item 2 and item 3

A correlated three-factor model showed an acceptable fit considering CFI and SRMR [χ2(63) = 286.6, CFI = 0.909, TLI = 0.885, RMSEA = 0.085, SRMR = 0.058]. The model was superior to the one-factor model (Δχ2 = 51.5, Δ*df* = 2, *p* < 0.001), however, it was inferior to the modified one-factor model (ΔBIC = -56). We further tested a modified three-factor model with residual covariance between items 2 and 3 which showed an acceptable fit to the data based on CFI and TLI and a good fit based on RMSEA and SRMR [χ2(62) = 191.7, CFI = 0.947, TLI = 0.932, RMSEA = 0.066, SRMR = 0.046]. The model was superior to the three-factor model (Δχ2 = 97.1, Δ*df* = 1, *p* < 0.001) as well as to the modified one-factor model (Δχ2 = 50.9, Δ*df* = 3, *p* < 0.001). See Fig. 1 for a detailed illustration of the model.

Finally, we tested a bi-factor model with one general SOC factor and three specific factors (comprehensibility, manageability, meaningfulness), however, the model was not identified.

#### Alternative short versions of the SOC scale

We further tested the fit of alternative shorter versions of the SOC scale by systematically removing poorly performing items. In SOC-12, item 2 was excluded (“Has it happened in the past that you were surprised by the behavior of people whom you thought you knew well?”). This item measures comprehensibility, hence SOC-12 has even distribution of items for each dimension (i.e., comprehensibility, manageability, meaningfulness). Item 2 has previously been identified as problematic [6] and also in our sample it did not perform well in any of the fitted SOC-13 models (i.e., low factor loading and explained variance). A one-factor SOC-12 model showed an acceptable fit to the data based on CFI and TLI and a good fit based on RMSEA and SRMR [χ2(54) = 221.1, CFI = 0.927, RMSEA = 0.079, SRMR = 0.048]. A correlated three-factor model showed an acceptable fit based on CFI and TLI and a good fit based on RMSEA and SRMR [χ2(52) = 171.1, CFI = 0.948, TLI = 0.932, RMSEA = 0.069 SRMR = 0.043]. The model was superior to the one-factor model (Δχ2 = 50, Δ*df* = 3, *p* < 0.001). Bi-factor model was not identified.

In SOC-11, we removed item 3 (“Has it happened that people whom you counted on disappointed you?”), which measures manageability. The item had the lowest factor loading and the lowest explained variance in the one-factor SOC-12. A one-factor SOC-11 model showed a good fit to the data [χ2 (44) = 138.5, CFI = 0.955, TLI = 0.944, RMSEA = 0.066, SRMR = 0.038]. A correlated three-factor model was identified but not acceptable due to covariance between comprehensibility and manageability higher than 1 (i.e., Heywood case; 39).

In SOC-10, we removed item 1 (“Do you have the feeling that you don’t really care about what goes on around you?”), which measures meaningfulness. The item had the lowest factor loading and the lowest explained variance in one-factor SOC-11. A one-factor SOC-10 model showed a good fit to the data [χ2 (35) = 126.6, CFI = 0.956, TLI = 0.943, RMSEA = 0.072, SRMR = 0.039]. As in the case of SOC-11, a correlated three-factor model was identified but not acceptable due to covariance between comprehensibility and manageability higher than 1.

**Fig. 2:**
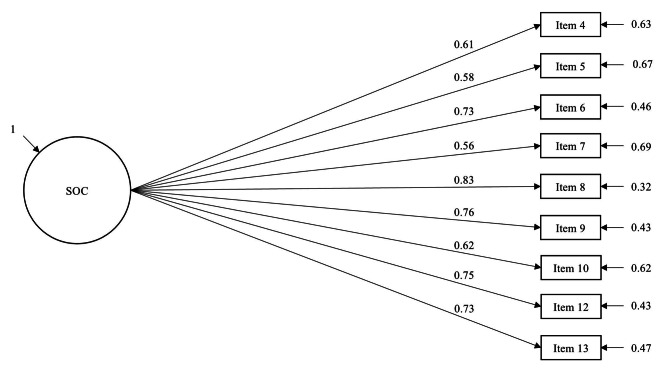
One-factor model of SOC-9

Finally, in SOC-9, we removed item 11 (“When something happened, have you generally found that… you overestimated or underestimated its importance / you saw the things in the right proportion”), which measures comprehensibility. The item had the lowest factor loading and the lowest explained variance in one-factor SOC-10. SOC-9 has an even distribution of three items for each dimension. A one-factor model showed a good fit to the data [χ2 (27) = 105.6, CFI = 0.959, TLI = 0.946, RMSEA = 0.076, SRMR = 0.038]. As in the previous models, a correlated three-factor model was identified but not acceptable due to covariance between comprehensibility and manageability higher than 1. See Fig. 2 for an illustration of one-factor SOC-9 model. Detailed results of the confirmatory factor analysis are shown in Table 2. In Table 3, we present the items of the SOC-13 (and SOC-9) scale with details about their facet structure.

**Table 2:** Model comparisons

Models		χ2 (df)	χ2/df	CFI	TLI	RMSEA	SRMR	BIC	ΔBIC	Δχ2	Δdf	M comparison
**SOC-13**												
M1	One-factor	338.2 (65)	5.20	0.889	0.867	0.092	0.062	22,844				
M2	One-factor with residual covariance	242.6 (64)	3.79	0.927	0.911	0.075	0.050	22,754	89	95.5***	1	M1 vs. **M2**
M3	Correlated three-factor	286.6 (62)	4.62	0.909	0.885	0.085	0.058	22,810	-56	-	2	**M2** vs. M3
M4	Correlated three-factor with residual covariance	191.7 (61)	3.14	0.947	0.932	0.066	0.046	22,722	32	50.9***	3	M2 vs. **M4**
**SOC-12** (- item 2)												
M5	One-factor	221.1 (54)	4.09	0.927	0.911	0.079	0.048	20,965				
M6	Correlated three-factor	171.1 (51)	3.35	0.948	0.932	0.069	0.043	20,934	31	50***	3	M5 vs. **M6**
**SOC-11** (- item 2,3)												
M7	One-factor	138.5 (44)	3.15	0.955	0.944	0.066	0.038	19,228	-	-	-	-
M8	Correlated three-factor	-	-	-	-	-	-	-	-	-	-	-
**SOC-10** (- item 2,3,1)												
M9	One-factor	126.6 (35)	3.61	0.956	0.943	0.072	0.039	17,276	-	-	-	-
M10	Correlated three-factor	-	-	-	-	-	-	-	-	-	-	-
**SOC-9** (- item 2,3,1,11)												
M11	One-factor	105.6 (27)	3.91	0.959	0.946	0.076	0.038	15,707	-	-	-	-
M12	Correlated three-factor	-	-	-	-	-	-	-	-	-	-	-

Note. Superior models are printed in bold; ***p < 0.001

**Table 3:**
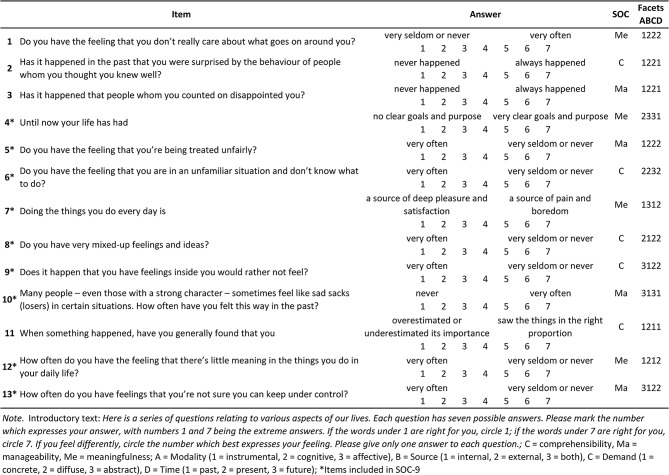
Items of the SOC-13 scale

### Differences by gender, age, and education

Correlation analysis indicated that SOC-13 increases with age (*r* = 0.32, *p* < 0.001), this finding was identical for all alternative short versions of the SOC scale (see Table 2). Further, the results of the two-tailed t-test showed that males (*M* = 4.8, *SD* = 1.08) had a significantly higher SOC-13 score [*t*(497) = 3.06, *p* = 0.002, *d* = 0.27] than females (*M* = 4.5, *SD* = 1.07). A one-way between-subjects ANOVA did not show any significant effect of level of education on SOC-13 score [F(2, 497) = 1.78, *p* = 0.169, η_p_^2^ = 0.022]. These results were similar for all alternative short versions of the SOC scale.

### Criterion validity

We found a moderately strong positive correlation (*r* = 0.61, *p* < 0.001) between SOC-13 and the positive mental health measure MHC, and a moderately strong negative correlation between SOC-13 and the negative mental health measure GAD (*r* = -0.68, *p* < 0.001). These findings were similar for all alternative short versions of the SOC scale (see Table 4).

**Table 4:** Correlation matrix with means, standard deviations and Cronbach alphas for each measure

	Min	Max	M	SD	alpha	1	2	3	4	5	6	7	8
1. SOC-13	1	7	4.63	1.08	0.88	1							
2. SOC-12	1	7	4.71	1.11	0.88	0.99	1						
3. SOC-11	1	7	4.78	1.14	0.88	0.98	0.99	1					
4. SOC-10	1	7	4.75	1.19	0.89	0.97	0.98	0.99	1				
5. SOC-9	1	7	4.78	1.25	0.89	0.97	0.98	0.99	1	1			
6. MHC-SF	1	6	3.82	0.96	0.90	0.61	0.62	0.62	0.61	0.62	1		
7. GAD-7	0	3	5.80	5.17	0.92	-0.68	-0.68	-0.68	-0.69	-0.69	-0.64	1	
8. Age	18	86	49.4	16.6	-	0.32	0.33	0.33	0.32	0.32	0.22	-0.28	1

Note. *N* = 498; all correlations are significant at *p* < 0.001

## Discussion

Our study examined the psychometric properties of the SOC-13 scale and its alternative short versions SOC-12, SOC-11, SOC-10, and SOC-9 in a representative sample of the Czech adult population. In line with existing studies [40], we found that SOC increases with age and that males score higher than females. In contrast to some prior findings [12], we did not find any significant differences in SOC based on the level of education. Further, we tested criterion validity using both positive and negative mental health outcomes (i.e., MHC and GAD). SOC had a strong positive correlation with MHC and a strong negative correlation with GAD, thus adding to the evidence about the criterion validity of the scale [6, 40].

Analysis of the factor structure showed that a one-factor SOC-13 had an inadequate fit to our data, however, an acceptable fit was achieved for a modified one-factor model with specified residual covariance between item 2 (“Has it happened in the past that you were surprised by the behavior of people whom you thought you knew well?”) and item 3 (“Has it happened that people whom you counted on disappointed you?”). A correlated three factor model with latent factors comprehensibility, manageability, and meaningfulness showed a better fit than the one factor-model. However, it was also necessary to specify residual covariance between item 2 and item 3 to reach an acceptable fit for all fit indices. A recent Slovenian study [41] found a similar result and several prior studies (see 6 for an overview) have noted that items 2 and 3 of the SOC-13 scale are problematic. Although the items pertain to different SOC dimensions (item 2 to comprehensibility, item 3 to manageability), multiple studies [e.g., 20, 42, 43] have reported moderately strong correlation between them and this is also the case in our study (*r* = 0.5, *p* < 0.001). The two items aptly illustrate the facet theory behind the scale construction as the SOC component represents only one building block of each item. Although items 2 and 3 theoretically pertain to different SOC components, they share the same elements from the other four facets (i.e., modality, source, demand, and time) which is reflected in the similarity of their wording. Therefore, they will necessarily share residual variance and this needs to be specified to achieve a good model fit. Drageset and Haugan [18] explain this similarity in that the people whom we know well are usually the ones that we count on, and feeling disappointed and surprised by the behavior of people we know well is closely related. Therefore, it should be theoretically justifiable to specify residual covariance between item 2 and item 3 as a possible solution to improve the fit. As we could show in our sample, the model fit significantly improved for both one-factor and three-factor solutions.

In addition, we examined the fit of alternative short versions of the SOC scale by systematically removing single items that performed poorly. First, in line with previous studies [6], we addressed the issue of residual covariance in SOC-13 by removing item 2, examining the factor structure of SOC-12. The remaining 12 items were equally distributed within the three SOC components with four items per each component. Interestingly, a one-factor model reached an acceptable fit and the fit further improved for a correlated three-factor model with latent factors of comprehensibility, manageability, and meaningfulness. Although correlated three-factor models were superior to one-factor models, we observed extreme covariances between latent variables, especially in case of comprehensibility and manageability (cov = 0.98). This suggests that the SOC components are not empirically separable and that, indeed, SOC is rather a one-dimensional global orientation with multiple components that are dynamically interrelated as Antonovsky proposed [2]. This notion was supported in a recent study that explored the dimensionality of the scale using a network perspective [16]. Our examination of SOC-11, SOC-10 and SOC-9 provided further support for a one-factor structure of the scale. All shorter versions yielded a good one-dimensional fit, however, we could not identify a correlated three-factor model fit due to the Heywood case. This refers to the situation when a solution that otherwise is satisfactory produces communality greater than one explained by the latent factor, which implies that the residual variance of the variable is negative [39]. In our case, this was true for the latent factors comprehensibility and manageability. However, we demonstrated that we could attain a good one-dimensional fit for all alternative short versions of SOC, and, importantly, they all showed comparable reliability and validity metrics to their longer counterpart SOC-13. In particular, SOC-9 shows very good fit indices and it performs equally well in validity analyses as SOC-13. Given these findings and existing evidence [5], we propose that future investigations may consider utilizing the SOC-9 scale instead of the SOC-13. It is interesting to point out that the majority of items that were removed for the shorter versions of the scale are negatively worded or reverse-scored (expect for item 11). This is in line with the latest research suggesting that such items can cause problems in model identification as they create additional method factors [44–46].

Finally, it is important to highlight that Antonovsky did not provide any information about the selection of the 13 items for the short version of the SOC scale [2]. For example, a detailed examination of the facet structure reveals that none of the items included in SOC-13 refers to *future* which is part of facet referring to time (i.e., past, present, future). Hence, considering the absence of explicit criteria for item selection in the SOC-13 scale, it would be interesting to gather data from diverse populations utilizing the full SOC-29 scale. Subsequently, through exploratory factor analysis, researchers could derive a new, theory- and empirical-driven, short version of the SOC scale.

### Strengths and limitations

A clear strength of our study is that our findings are based on a representative sample that accurately reflects the Czech adult population. Moreover, we implemented rigorous data cleaning procedures, meticulously excluding participants who provided potentially careless or low-quality responses. By doing so, we ensured that our conclusions are based on high-quality data and that they are generalizable to our target population of Czech adults. Finally, we conducted a thorough back-translation procedure to achieve an accurate Czech version of the SOC scale and we carried out systematic testing of different short versions of the SOC scale.

However, our study also has some limitations. First, our conclusions are based on data from a culturally specific country and they may not be generalizable to other populations. It is important to note, however, that most of our findings are in line with multiple existing studies which supports the validity of our conclusions. Second, the data were collected during a later stage of the COVID-19 pandemic, which may have impacted particularly the mental health outcomes we used for criterion validity. It would be worthwhile to investigate whether the data replicate in our population outside of this exceptional situation. Third, it should be noted that we did not examine test-retest reliability of the scale due to the cross-sectional design of our study. Finally, self-reported data are subject to common method biases such as social desirability, recall bias, or consistency motive [47]. We aimed to minimize this risk by implementing various strategies in the questionnaire, such as randomization of items and the use of disqualifying items (e.g. “Please, choose option number 2”) to disqualify careless answers.

## Conclusion

Our study contributes to decades of ongoing research on SOC, the main pillar of the theory of salutogenesis. In line with existing research, we found evidence for the validity of the SOC as a construct, but we could not identify a clear factorial structure of the SOC-13 scale. However, following Antonovsky’s conception of the scale, we believe it is theoretically sound to aim for a one-factor solution of the scale and we could show that this is possible with shorter versions of the SOC scale. We particularly recommend using the SOC-9 scale in future research which shows an excellent one-factor fit and validity indices comparable to SOC-13. Finally, since Antonovsky does not explain how he selected the items of the SOC-13 scale, it would be interesting to examine the possibility of developing a new one-dimensional short version based on exploratory factor analysis of the original SOC-29 scale.

### Electronic supplementary material

Below is the link to the electronic supplementary material.

Supplementary Material 1

Supplementary Material 2

Supplementary Material 3

## Data Availability

The datasets used and analyzed during the current study and the R code used for the statistical analysis are available as supplementary material.

## Footnotes

1 www.czso.cz.
