# Supplementary material for: The sense of coherence scale: psychometric properties in a representative sample of the Czech adult population
Source: BMC Psychol. 2024 May 26;12:293. doi: 10.1186/s40359-024-01805-7 (PMC11128106; doi:10.1186/s40359-024-01805-7)
Supplement: Supplementary file 2 — Supplementary Material 2 [file 40359_2024_1805_MOESM2_ESM.pdf]

Orientation to life questionnaire: SOC-29 and SOC-13

Here is a series of questions relating to various aspects of our lives. Each question has seven possible answers. Please mark the number which expresses your answer, with numbers 1 and 7 being the extreme answers. If the words under 1 are right for you, circle 1; if the words under 7 are right for you, circle 7. If you feel differently, circle the number which best expresses your feeling. Please give only one answer to each question.

			Item	Answering format (1-7)			SOC component	Facets (ABCD)	Reverse-scored
				1	2 3 4 5 6	7			
		1	Do you have the feeling that you don't really care about what goes on around you?	very seldom or never		very often	Me	1222	x
		2	Has it happened in the past that you were surprised by the behaviour of people whom you thought you knew well?	never happened		always happened	C	1221	x
		3	Has it happened that people whom you counted on disappointed you?	never happened		always happened	Ma	1221	x
		4	Until now your life has had	no clear goals and purpose		very clear goals and purpose	Me	2331	
		5	Do you have the feeling that you're being treated unfairly?	very often		very seldom or never	Ma	1222	
		6	Do you have the feeling that you are in an unfamiliar situation and don't know what to do?	very often		very seldom or never	C	2232	
	SOC-13	7	Doing the things you do every day is	a source of deep pleasure and satisfaction		a source of pain and boredom	Me	1312	x
		8	Do you have very mixed-up feelings and ideas?	very often		very seldom or never	C	2122	
		9	Does it happen that you have feelings inside you would rather not feel?	very often		very seldom or never	C	3122	
		10	Many people – even those with a strong character – sometimes feel like sad sacks (losers) in certain situations. How often have you felt this way in the past?	never		very often	Ma	3131	x
		11	When something happened, have you generally found that	you overestimated or underestimated its importance		you saw the things in the right proportion	C	1211	
		12	How often do you have the feeling that there's little meaning in the things you do in your daily life?	very often		very seldom or never	Me	1212	
		13	How often do you have feelings that you're not sure you can keep under control?	very often		very seldom or never	Ma	3122	
		14	When you talk to people, do you have the feeling that they don't understand you?	never		always have this feelling	C	1312	x
		15	In the past, when you had to do something which depended upon cooperation with others, did you have the feeling that it	surely woulnd't get done		surely would get done	Ma	1111	
		16	Think of the people with whom you come into contact daily, aside from the ones to whom you feel closest. How well do you know most of them?	you feel that they're strangers		you know them very well	C	1322	
SOC-29		17	Life is	full of interest		completely routine	Me	2332	x
		18	In the past ten years your life has been	full of changes without your knowing what will happen next		completely consistent and clear	C	2331	
		19	Most of the things you do in the future will probably be	completely fascinating		deadly boring	Me	1313	x
		20	What best describes how you see life	one can always find a solution to painful things in life		there is no solution to painful things in life	Ma	2332	x
		21	When you think about your life, you very often	feel how good it is to be alive		ask yourself why you exist at all	Me	2132	x
		22	When you face a difficult problem, the choice of a solution is	always confusing and hard to find		always completely clear	C	1112	
		23	Your life in the future will probably be	full of changes without your knowing what will happen next		completely consistent and clear	C	2333	
		24	When something unpleasant happened in the past your tendency was	"to eat yourself up"		to say "ok that's that, I have to live with it" and go on	Ma	3211	
		25	When you do something that gives you a good feeling	it's certain that you'll go on feeling good		it's certain that something will happen to spoil the feeling	Ma	1113	x
		26	You anticipate that your personal life in the future will be	totally without meaning and purpose		full of meaning and purpose	Me	2333	
		27	Do you think that there will always be people whom you'll be able to count on in the future?	you're certain there will be		you doubt there will be	Ma	1223	x
		28	Does it happen that you have the feeling that you don't know exactly what's about to happen?	very often		very seldom or never	C	2233	
		29	When you think of the difficulties you are likely to face in important aspects of your life, do you have the feeling that	you will always succeed in overcoming the difficulties		you won't succeed in overcoming the difficulties	Ma	1313	x

Note. **SOC component:** C = comprehensibility (11 items), Ma = manageability (10 items), Me = meaningfulness (8 items); **Facets:** A) Modality (1 = instrumental, 2 = cognitive, 3 = affective), B) Source (1 = internal, 2 = external, 3 = both), C) Demand (1 = concrete, 2 = diffuse, 3 = abstract), D) Time (1 = past, 2 = present, 3 = future); **Reverse-scored:** 13 items.

Orientation to life questionnaire: SOC-29 a SOC-13

Zde je řada otázek týkajících se různých aspektů našeho života. Každá otázka má sedm možných odpovědí. Označte prosím číslo, které vyjadřuje Vaši odpověď, přičemž čísla 1 a 7 jsou krajní odpovědi. Pokud pro Vás platí odpověď pod číslem 1, zakroužkujte 1; pokud pro Vás platí odpověď pod číslem 7, zakroužkujte 7. Pokud máte jiný pocit, zakroužkujte číslo, které nejlépe vyjadřuje Váš pocit. Na každou otázku uveďte pouze jednu odpověď.

			Položka	Odpověď (1-7)							SOC	Facets	Reverse-
				1	2	3	4	5	6	7	component	(ABCD)	scored
SOC-29	SOC-13	1	Máte pocit, že se v podstatě nezajímáte o to, co se kolem vás děje?	zřídka nebo nikdy						často	Me	1222	x
		2	Stalo se vám v minulosti, že jste byl(a) překvapen(a) chováním lidí, o nichž jste si myslel(a), že je dobře znáte?	nikdy se mi to nestalo						vždy se mi to stalo	C	1221	x
		3	Stalo se vám, že vás zklamali lidé, kterým jste důvěřoval(a) a s nimiž jste počítal(a)?	nikdy se mi to nestalo						vždy se mi to stalo	Ma	1221	x
		4	Váš život doposud	neměl jasný cíl a smysl						měl velmi jasný cíl a smysl	Me	2331	
		5	Míváte pocit, že se s vámi zachází nespravedlivě?	velmi často						zřídka nebo nikdy	Ma	1222	
		6	Míváte pocit, že jste v neznámé situaci a nevíte dost dobře, co dělat?	velmi často						zřídka nebo nikdy	C	2232	
		7	Vaše každodenní činnosti jsou pro Vás	zdrojem hluboké radosti a uspokojení						zdrojem utrpení a nudy	Me	1312	x
		8	Míváte velmi nejasné představy a smíšené pocity?	velmi často						zřídka nebo nikdy	C	2122	
		9	Stává se vám, že v sobě máte pocity, které byste raději neměl(a)?	velmi často						zřídka nebo nikdy	C	3122	
		10	Mnozí lidé, i ti kdo mají pevný charakter, se někdy cítí poražení a smutní. Jak často jste se takto cítil(a) v minulosti vy?	zřídka nebo nikdy						velmi často	Ma	3131	x
		11	Když došlo k nějaké události, obvykle jste zjistil(a), že	jste její důležitost přecenil(a) nebo podcenil(a)						jste její důležitost posoudil(a) správně	C	1211	
		12	Jak často míváte pocit, že to, co děláte ve svém běžném životě, nemá žádný smysl?	velmi často						zřídka nebo nikdy	Me	1212	
		13	Jak často míváte pocity, u kterých si nejste jist(a), že je dokážete udržet pod kontrolou?	velmi často						zřídka nebo nikdy	Ma	3122	
		14	Když mluvíte s lidmi, máte pocit, že vám nerozumějí?	nikdy						vždy mám ten pocit	C	1312	x
		15	Když jste v minulosti měl(a) dělat něco, kde byla nezbytná spolupráce s druhými lidmi, měl(a) jste pocit	že se to určitě nezvládne						že se to určitě zvládne	Ma	1111	
		16	Vzpomeňte si na lidi, s nimiž denně přicházíte do kontaktu, mimo členy své vlastní rodiny. Jak dobře většinu z nich znáte?	připadá mi, že je vůbec neznám						znám je velmi dobře	C	1322	
		17	Život je	velmi zajímavý						zcela rutinní	Me	2332	x
		18	V uplynulých deseti letech Váš život byl	plný změn, kdy jste nikdy netušil(a), co se bude dít dál						zcela konzistentní a zřejmý	C	2331	
		19	Většina věcí, které v budoucnu budete dělat bude nejspíš	velice vzrušujících						velice nudných	Me	1313	x
		20	Co nejlépe vystihuje, jak Vy sám/sama vnímáte život?	člověk vždy může najít řešení obtížných životních situací						neexistuje žádné řešení obtížných životních situací	Ma	2332	x
		21	Když přemýšíte nad svým životem, pak velmi často	cítíte, jak je dobré být naživu						ptáte se sám/sama sebe, proč vůbec existujete	Me	2132	x
		22	Když stojíte před obtížným problémem, pak se Vám jeho řešení zdá	vždy složité a těžko se hledá						vždy zcela jasné	C	1112	
		23	Váš budoucí život bude pravděpodobně	plný změn, kdy sám/sama ani nebudete tušit, co se bude dít dál						zcela konzistentní a zřejmý	C	2333	
		24	Když se Vám v minulosti stalo něco nepříjemného, měl(a) jste tendenci	užírat se tím						říci si "stalo se, musím s tím žít" a jít dál	Ma	3211	
		25	Když děláte něco, z čeho máte dobrý pocit, je jisté, že	se tak dobře budete cítit i nadále						se stane něco, co Vám ten dobrý pocit pokazí	Ma	1113	x
		26	Očekáváte, že Váš budoucí osobní život bude	zcela neužitečný a bezesmyslný						velmi užitečný a smysluplný	Me	2333	
		27	Domníváte se, že kolem Vás v budoucnosti vždy budou lidé, na které se budete moci spolehnout?	jste si jist(a), že budou takoví lidé						pochybujete, že budou takoví lidé	Ma	1223	x
		28	Stává se Vám, že máte pocit, kdy nevíte dost dobře, co se bude dít dále?	velmi často se mi to stává						stává se mi to velmi zřídka nebo nikdy	C	2233	
		29	Když pomyslíte na obtíže, s nimiž se pravděpodobně setkáte v důležitých aspektech svého života, máte pocit že	se Vám vždy podaří obtíže překonat						se Vám nepodaří obtíže překonat	Ma	1313	x

Note. **SOC component:** C = comprehensibility (11 items), Ma = manageability (10 items), Me = meaningfulness (8 items); **Facets:** A) Modality (1 = instrumental, 2 = cognitive, 3 = affective), B) Source (1 = internal, 2 = external, 3 = both), C) Demand (1 = concrete, 2 = diffuse, 3 = abstract), D) Time (1 = past, 2 = present, 3 = future); **Reverse-scored:** 13 items.
