# Supplementary material for: The sense of coherence scale: psychometric properties in a representative sample of the Czech adult population
Source: BMC Psychol. 2024 May 26;12:293. doi: 10.1186/s40359-024-01805-7 (PMC11128106; doi:10.1186/s40359-024-01805-7)
Supplement: Supplementary file 3 — Supplementary Material 3 [file 40359_2024_1805_MOESM3_ESM.pdf]

The Sense of Coherence Scale (SOC-13): Psychometric Properties in a Representative Sample of the Czech Adult Population

Anonymous

2024-01-25

```
library(tidyverse)
library(psych)
library(forcats)
library(lavaan)
library(skimr)
library(corrplot)
library(car)
library(RColorBrewer)
library(sjPlot)
library(parameters)
library(Hmisc)


df <- read_csv("1_data/data_cleaned_soc.csv")
```

Confirmatory Factor Analysis

```
### SOC-13 ###
# Model specification - One-factor model
model <- '
          # Defining latent variables
          socs =~ soc1 + soc2 + soc3 + soc4 + soc5 + soc6 + soc7 + soc8 + soc9 + soc10 + soc11 + soc12 +
'

#  Model estimation
fit1 <- cfa(model,
            data = df,
            estimator = "ML")

summary(fit1,
        rsquare = TRUE,
        standardized = TRUE,
        fit.measures = TRUE)

# Model specification - One-factor model (Residual covariance)
model <- '
          # Defining latent variables
          socs =~ soc1 + soc2 + soc3 + soc4 + soc5 + soc6 + soc7 + soc8 + soc9 + soc10 + soc11 + soc12 +

          soc2 ~~ soc3
```

```
'

#  Model estimation
fit2 <- cfa(model,
            data = df,
            estimator = "ML")

summary(fit2,
        rsquare = TRUE,
        standardized = TRUE,
        fit.measures = TRUE)

modindices(fit2, sort. = TRUE)


# Model specification - Three-factor model
model <- '
          # Defining latent variables
          me =~ soc1 + soc4 + soc7 + soc12
          ma =~ soc3 + soc5 + soc10 + soc13
          co =~ soc2 + soc6 + soc8 + soc9 + soc11
'

#  Model estimation
fit3 <- cfa(model,
            data = df,
            estimator = "ML")

summary(fit3,
        rsquare = TRUE,
        standardized = TRUE,
        fit.measures = TRUE)

# Model specification - Three-factor model (Residual covariance)
model <- '
          # Defining latent variables
          me =~ soc1 + soc4 + soc7 + soc12
          ma =~ soc3 + soc5 + soc10 + soc13
          co =~ soc2 + soc6 + soc8 + soc9 + soc11

          soc2 ~~ soc3
'

#  Model estimation
fit4 <- cfa(model,
            data = df,
            estimator = "ML")

summary(fit4,
        rsquare = TRUE,
        standardized = TRUE,
        fit.measures = TRUE)
```

```
modindices(fit5, sort. = TRUE)

# Model specification - Bi-factor model
model <- '
          # Defining latent variables
          me =~ soc1 + soc4 + soc7 + soc12
          ma =~ soc3 + soc5 + soc10 + soc13
          co =~ soc2 + soc6 + soc8 + soc9 + soc11
          socs =~ soc1 + soc2 + soc3 + soc4 + soc5 + soc6 + soc7 + soc8 + soc9 + soc10 + soc11 + soc12 +

          # Setting orthogonal (uncorrelated) factors
          me ~~ 0*ma
          me ~~ 0*co
          ma ~~ 0*co
'

#  Model estimation
fit5 <- cfa(model,
            data = df,
            estimator = "ML",
            std.lv = TRUE)

summary(fit5,
        rsquare = TRUE,
        standardized = TRUE,
        fit.measures = TRUE)

bifactorIndices(fit4, UniLambda = fit1)

anova(fit1, fit2)
BIC(fit1) - BIC(fit2)
anova(fit2, fit3)
BIC(fit2) - BIC(fit3)
anova(fit2, fit4)
BIC(fit2) - BIC(fit4)
anova(fit5, fit6)
BIC(fit5) - BIC(fit6)

############
## SOC-12 ## - item 2
############
## Calculating means
# SOC-12
df$soc_12 <- rowMeans(df[,c("soc1", "soc3", "soc4", "soc5", "soc6", "soc7", "soc8", "soc9", "soc10", "s

# Comprehensibility
df$soc_co <- rowMeans(df[,c("soc6", "soc8", "soc9", "soc11")], na.rm = TRUE)

# Manageability
df$soc_ma <- rowMeans(df[,c("soc3", "soc5", "soc10", "soc13")], na.rm = TRUE)

# Meaningfulness
df$soc_me <- rowMeans(df[,c("soc1", "soc4", "soc7", "soc12")], na.rm = TRUE)
```

```r
### Correlation matrix
soc12 <- df %>%
  dplyr::select(soc1, soc3, soc4, soc5, soc6, soc7, soc8, soc9, soc10, soc11, soc12, soc13)

soc_co <- df %>%
  dplyr::select(soc6, soc8, soc9, soc11)

soc_ma <- df %>%
  dplyr::select(soc3, soc5, soc10, soc13)

soc_me <- df %>%
  dplyr::select(soc1, soc4, soc7, soc12)

soc <- df %>%
  dplyr::select(soc_12, soc_co, soc_ma, soc_me)

mat <- cor(soc, method = "pearson", use = "complete.obs")
mat
corrplot(mat, method = "number", type = "lower", tl.col = "black")
rcorr(as.matrix(soc), type="pearson")
skim(soc)

### Reliability
# Cronbach alpha
alpha(soc12)
alpha(soc_co)
alpha(soc_ma)
alpha(soc_me)

## Convergent validity
val <- df %>%
  dplyr::select(soc_12, gad, mhc)

mat <- cor(val, method = "pearson", use = "complete.obs")
corrplot(mat, method = "number", type = "upper", tl.col = "black")
round(mat, 2)

## Differences by age, gender, education
cor(df$soc_12, df$age)

t.test(soc_12 ~ gender, data = df)

a <- aov(soc ~ edu, data = df)
summary(a)
TukeyHSD(a)

### CFA ###
# Model specification - One-factor model
model <- '
          # Defining latent variables
          socs =~ soc1 + soc3 + soc4 + soc5 + soc6 + soc7 + soc8 + soc9 + soc10 + soc11 + soc12 + soc13
'
```

```
#  Model estimation
fit6 <- cfa(model,
            data = df,
            estimator = "ML",
            std.lv = TRUE)

summary(fit6,
        rsquare = TRUE,
        standardized = TRUE,
        fit.measures = TRUE)

# Model specification - Correlated three-factor model
model <- '
          # Defining latent variables
          me =~ soc1 + soc4 + soc7 + soc12
          ma =~ soc3 + soc5 + soc10 + soc13
          co =~ soc6 + soc8 + soc9 + soc11
'

#  Model estimation
fit7 <- cfa(model,
            data = df,
            estimator = "ML")

summary(fit7,
        rsquare = TRUE,
        standardized = TRUE,
        fit.measures = TRUE)

### Model testing
anova(fit6, fit7)
BIC(fit6) - BIC(fit7)
anova(fit9, fit10)
BIC(fit9) - BIC(fit10)
anova(fit9, fit11)
BIC(fit9) - BIC(fit11)

############
## SOC-11 ## - item 2,3
############
## Calculating means
# SOC-11
df$soc_11 <- rowMeans(df[,c("soc1", "soc4", "soc5", "soc6", "soc7", "soc8", "soc9", "soc10", "soc11", "[illegible]

# Comprehensibility
df$soc_co <- rowMeans(df[,c("soc6", "soc8", "soc9", "soc11")], na.rm = TRUE)

# Manageability
df$soc_ma <- rowMeans(df[,c("soc5", "soc10", "soc13")], na.rm = TRUE)

# Meaningfulness
df$soc_me <- rowMeans(df[,c("soc1", "soc4", "soc7", "soc12")], na.rm = TRUE)
```

```r
### Correlation matrix
soc11 <- df %>%
  dplyr::select(soc1, soc4, soc5, soc6, soc7, soc8, soc9, soc10, soc11, soc12, soc13)

mat <- cor(soc, method = "pearson", use = "complete.obs")
mat
corrplot(mat, method = "number", type = "lower", tl.col = "black")
rcorr(as.matrix(soc), type="pearson")
skim(soc)

### Reliability
# Cronbach alpha
alpha(soc11)
alpha(soc_co)
alpha(soc_ma)
alpha(soc_me)

## Convergent validity
val <- df %>%
  dplyr::select(soc_11, gad, mhc)

mat <- cor(val, method = "pearson", use = "complete.obs")
corrplot(mat, method = "number", type = "upper", tl.col = "black")
round(mat, 2)

## Differences by age, gender, education
cor(df$soc_11, df$age)

t.test(soc_11 ~ gender, data = df)

a <- aov(soc_11 ~ edu, data = df)
summary(a)
TukeyHSD(a)

### CFA ###
# Model specification - One-factor model
model <- '
          # Defining latent variables
          socs =~ soc1 + soc4 + soc5 + soc6 + soc7 + soc8 + soc9 + soc10 + soc11 + soc12 + soc13
'

#  Model estimation
fit6 <- cfa(model,
            data = df,
            estimator = "ML",
            std.lv = TRUE)

summary(fit6,
        rsquare = TRUE,
        standardized = TRUE,
        fit.measures = TRUE)

# Model specification - Correlated three-factor model
```

```
model <- '
          # Defining latent variables
          me =~ soc1 + soc4 + soc7 + soc12
          ma =~ soc5 + soc10 + soc13
          co =~ soc6 + soc8 + soc9 + soc11
'

#  Model estimation
fit7 <- cfa(model,
            data = df,
            estimator = "ML")

summary(fit7,
        rsquare = TRUE,
        standardized = TRUE,
        fit.measures = TRUE)

############
## SOC-10 ## - item 1,2,3
############
## Calculating means
# SOC-10
df$soc_10 <- rowMeans(df[,c("soc4", "soc5", "soc6", "soc7", "soc8", "soc9", "soc10", "soc11", "soc12", [illegible]

### Correlation matrix
soc10 <- df %>%
  dplyr::select(soc4, soc5, soc6, soc7, soc8, soc9, soc10, soc11, soc12, soc13)

mat <- cor(soc, method = "pearson", use = "complete.obs")
mat
corrplot(mat, method = "number", type = "lower", tl.col = "black")
rcorr(as.matrix(soc), type="pearson")
skim(soc)

### Reliability
# Cronbach alpha
alpha(soc10)
alpha(soc_co)
alpha(soc_ma)
alpha(soc_me)

## Convergent validity
val <- df %>%
  dplyr::select(soc_10, gad, mhc)

mat <- cor(val, method = "pearson", use = "complete.obs")
corrplot(mat, method = "number", type = "upper", tl.col = "black")
round(mat, 2)

## Differences by age, gender, education
cor(df$soc_10, df$age)

t.test(soc_10 ~ gender, data = df)
```

```r
a <- aov(soc_10 ~ edu, data = df)
summary(a)
TukeyHSD(a)

### CFA ###
# Model specification - One-factor model
model <- '
          # Defining latent variables
          socs =~ soc4 + soc5 + soc6 + soc7 + soc8 + soc9 + soc10 + soc11 + soc12 + soc13
'

#  Model estimation
fit6 <- cfa(model,
            data = df,
            estimator = "ML",
            std.lv = TRUE)

summary(fit6,
        rsquare = TRUE,
        standardized = TRUE,
        fit.measures = TRUE)

# Model specification - Correlated three-factor model
model <- '
          # Defining latent variables
          me =~ soc4 + soc7 + soc12
          ma =~ soc5 + soc10 + soc13
          co =~ soc6 + soc8 + soc9 + soc11
'

#  Model estimation
fit7 <- cfa(model,
            data = df,
            estimator = "ML")

summary(fit7,
        rsquare = TRUE,
        standardized = TRUE,
        fit.measures = TRUE)

### Model testing
anova(fit6, fit7)
BIC(fit6) - BIC(fit7)
anova(fit9, fit10)
BIC(fit9) - BIC(fit10)
anova(fit9, fit11)
BIC(fit9) - BIC(fit11)

############
## SOC-9 ## - item 1,2,3,11
############
## Calculating means
# SOC-9
```

```r
df$soc_9 <- rowMeans(df[,c("soc4", "soc5", "soc6", "soc7", "soc8", "soc9", "soc10", "soc12", "soc13")],

# Comprehensibility
df$soc_co <- rowMeans(df[,c("soc6", "soc8", "soc9")], na.rm = TRUE)

# Manageability
df$soc_ma <- rowMeans(df[,c("soc5", "soc10", "soc13")], na.rm = TRUE)

# Meaningfulness
df$soc_me <- rowMeans(df[,c("soc4", "soc7", "soc12")], na.rm = TRUE)

### Correlation matrix
soc9 <- df %>%
  dplyr::select(soc4, soc5, soc6, soc7, soc8, soc9, soc10, soc12, soc13)

soc_co <- df %>%
  dplyr::select(soc6, soc8, soc9)

soc_ma <- df %>%
  dplyr::select(soc5, soc10, soc13)

soc_me <- df %>%
  dplyr::select(soc4, soc7, soc12)

soc <- df %>%
  dplyr::select(soc_9, soc_co, soc_ma, soc_me)

mat <- cor(soc, method = "pearson", use = "complete.obs")
mat
corrplot(mat, method = "number", type = "lower", tl.col = "black")
rcorr(as.matrix(soc), type="pearson")
skim(soc)

### Reliability
# Cronbach alpha
alpha(soc9)
alpha(soc_co)
alpha(soc_ma)
alpha(soc_me)

## Differences by age, gender, education
cor(df$soc_9, df$age)

t.test(soc_9 ~ gender, data = df)

a <- aov(soc_9 ~ edu, data = df)
summary(a)
TukeyHSD(a)

### CFA ###
# Model specification - One-factor model
model <- '
          # Defining latent variables
```

```r
         socs =~ soc4 + soc5 + soc6 + soc7 + soc8 + soc9 + soc10 + soc12 + soc13
'

#  Model estimation
fit6 <- cfa(model,
            data = df,
            estimator = "ML",
            std.lv = TRUE)

summary(fit6,
        rsquare = TRUE,
        standardized = TRUE,
        fit.measures = TRUE)

# Model specification - Correlated three-factor model
model <- '
          # Defining latent variables
          me =~ soc4 + soc7 + soc12
          ma =~ soc5 + soc10 + soc13
          co =~ soc6 + soc8 + soc9
'

#  Model estimation
fit7 <- cfa(model,
            data = df,
            estimator = "ML")

summary(fit7,
        rsquare = TRUE,
        standardized = TRUE,
        fit.measures = TRUE)


## Convergent validity all versions
val <- df %>%
  dplyr::select(soc, soc_12, soc_11, soc_10, soc_9, soc_6, soc_3, mhc, gad, age)

mat <- cor(val, method = "pearson", use = "complete.obs")
corrplot(mat, method = "number", type = "upper", tl.col = "black")
round(mat, 2)
skim(val)
table(df$age)
```
